# The impact of deep learning and omics data in transforming precision therapy for brain cancer

**DOI:** 10.3389/fbinf.2026.1811813

**Published:** 2026-09-22

**Authors:** Sana Munquad, Anam Upadhyay, Asim Bikas Das

**Affiliations:** Department of Biotechnology, National Institute of Technology Warangal, Warangal, India

**Keywords:** brain cancer, copy number alteration (CNA), deep learning (DL), DNA methylation, gene expression, mutation, omics

## Abstract

The combination of AI with high-throughput genomics has revolutionized oncology, particularly in the case of brain cancer, a diagnostically complicated and heterogeneous tumor. With the explosion of omics data and computational power, deep learning (DL) has evolved as a powerful computational approach for the interpretation of the molecular topography of brain tumors. Utilizing mono-omics and multi-omics data, including gene expression, somatic mutations, DNA methylation, copy number alterations, and miRNA profiles, deep learning algorithms can detect complex, nonlinear patterns underlying tumor biology and clinical behavior. Several DL architectures have been used in recent studies for classification, biomarker discovery, and subtype prediction in brain cancer. The combination of multi-omics data has been especially useful, since oncogenic changes arise at several molecular levels; therefore, the omission of any specific omics aspect might undermine diagnostic accuracy and therapeutic prediction. Here, in this review, DL-based approaches to various omics modalities are reviewed, covering the tools constructed, architectures, and performance levels, and then delving into integrative multi-omics frameworks that maximize the accuracy and interpretability of diagnostic models. It also discusses the significance of AI-based approaches in advancing personalized treatment for brain cancer, emphasizing their capacity to predict patient-specific drug responses. Notwithstanding these breakthroughs, some major challenges remain, which include data heterogeneity, explainability of models, computational requirements, and ethical issues related to the use of genomic data. Together, the review highlights the potential of DL and multi-omics integration in advancing precision oncology for brain cancer by enhancing diagnostic precision, prognostic accuracy, and personalized therapy development.

## 1 Introduction

Although rare, brain tumors contribute significantly to mortality and morbidity across all age groups. Despite intense research that has spanned decades to understand the causes of brain tumors, no significant risk factor has emerged to explain a large proportion of these cases. These tumors are characterized by their histological complexity, with the World Health Organization’s International Classification of Diseases for Oncology categorizing over 100 different types (Louis et al., 2021). Of all these, the most common subtype of brain cancer which arises from the gliocytes of the brain are gliomas. Gliomas are also graded from Grades I to IV. Grade I gliomas are of low proliferative behavior and tend to recur and grow into higher-grade lesions. Glioblastoma multiforme (GBM) is the most aggressive, invasive, and undifferentiated type of tumor, and it is classified as Grade IV by the WHO (Oszvald et al., 2012). The molecular heterogeneity of brain cancer is a significant challenge for accurate diagnosis and effective therapy. Tumor characteristics such as mutation, gene expression, epigenetics, and protein profiles, are extraordinarily diverse. Due to such huge diversity, identification of the kind of brain tumor by conventional diagnostic methods, such as histological examination, is challenging, subject to intra-observer and inter-observer variability, and can result in poor clinical outcomes. Moreover, the patient’s life expectancy varies depending on the type of tumor; for example, the life expectancy of GBM patients is the lowest of all tumor types (Tamimi and Juweid, 2017). Therefore, knowing the best form of treatment, such as surgery or chemotherapy, is dependent upon the recognition of subtypes and grades and the prediction of drug response for a particular patient.

It is now understood that molecular alterations contributing to an increased risk of brain cancer are scattered all over the genome (Haque et al., 2011). Thus, it is essential to analyze genome-wide data or omics data to capture the molecular landscape of various forms of brain cancer. Most of the biomarkers for brain cancer up until this point have been discovered using inconclusive, low-throughput techniques without taking omics data into account (Shalaby et al., 2016). The vast majority of observations from low-throughput experiments cannot, however, offer therapeutic answers, as they cannot provide an overall view of the complex systems of cancer. These include accurately pinpointing the subtypes and grades and patient-specific drug response prediction, which are very important for determining the best mode of treatment, such as surgery or chemotherapy. This requires the scope of research to be on omics data analysis, intending to properly capture complex relationships of multiple layers of omics, which include genomics, transcriptomics, proteomics, metabolomics, and epigenomics (Figure 1). The size of this omics data has been exponentially increasing in nature due to advancements in high-throughput technologies. Hence, powerful computing approaches are needed to extract new insights from enormous amounts of data.

**FIGURE 1:**
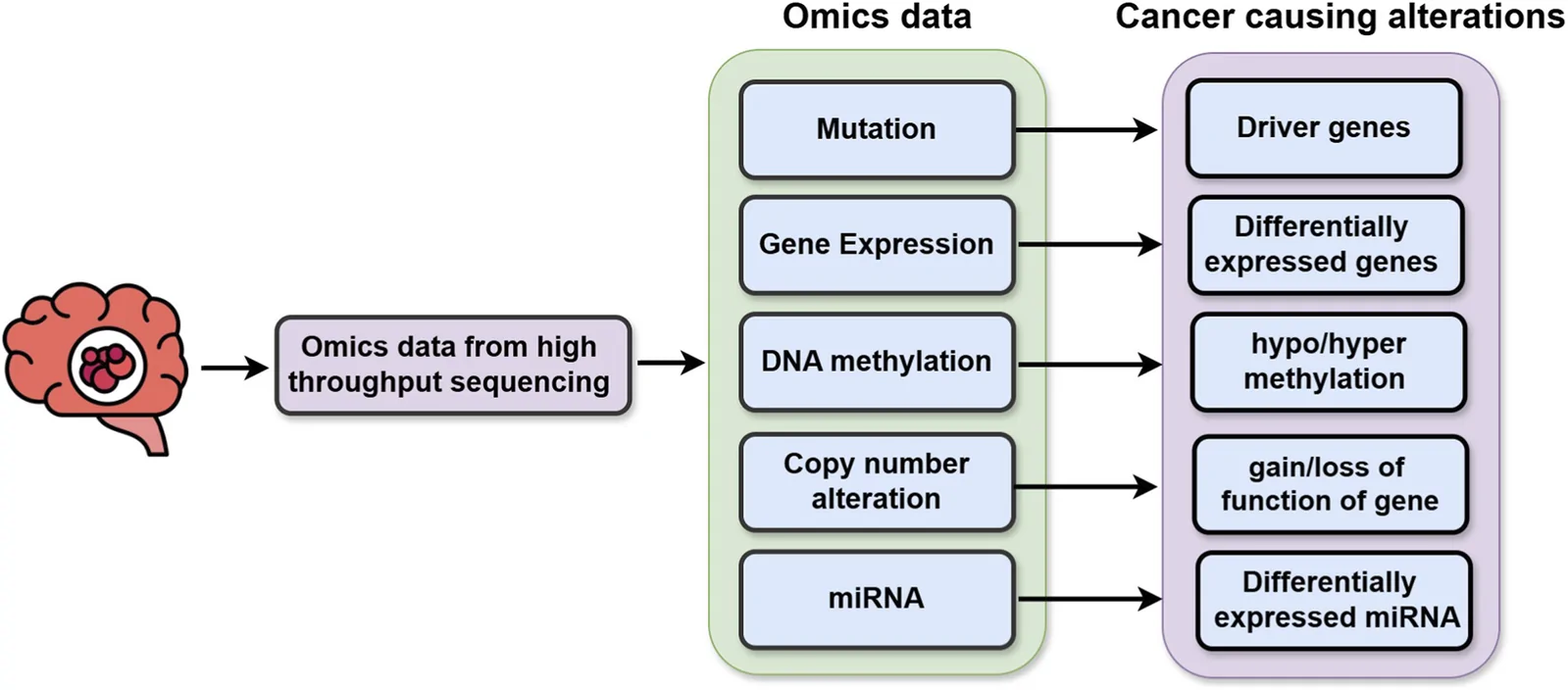
Mapping cancer-associated alterations in glioma from omics data. High-throughput sequencing generates multiple layers of omics data, including somatic mutations, gene expression, DNA methylation, copy number alterations (CNAs), and miRNA expression. The analysis of these data identifies key cancer-associated alterations, which provide a comprehensive molecular landscape of glioma.

Artificial intelligence (AI) has become an essential tool in improving tumor detection, diagnosis, treatment, and prevention, contributing significantly to advancements in the overall care of cancer patients (Hunter et al., 2022). Machine learning (ML) and DL have, no doubt, revolutionized several neuropathological practices. These technologies assist in the major processes: data preprocessing, feature extraction, feature selection, dimensionality reduction, classification, and personalized treatment planning.

In addition to diagnosis, AI plays a crucial role in enhancing treatment strategies, enabling more accurate predictions of patient responses to specific therapies (Taherdoost and Ghofrani, 2024). It also helps identify potential preventive measures by analyzing large datasets to detect early signs or genetic predispositions to brain cancer (Abdusalomov et al., 2023). DL algorithms have emerged as one of the most successful techniques because of their capacity for handling high-dimensionality, effective data integration, efficient dimensionality reduction, stability, and higher prediction accuracy (Sarker, 2021). Furthermore, the DL algorithm is being used in cancer genomics for personalized medicine because of its superior efficiency in capturing nonlinear information from heterogeneous datasets (Kang et al., 2022). DL is also making advances in the classification, diagnosis, and prognosis of brain cancer (ZainEldin et al., 2022). Early diagnosis of the brain tumor is challenging because it is often non-symptomatic. In most cases, it results in late diagnosis and denies patients timely treatment, which raises the death risk for patients (Ozawa et al., 2018). Thus, there is a growing need to discover new ways of diagnosis and treatment, such as the stage and subtype identification of brain cancer, or even design drugs that can at least decrease the death chances through wrong treatment selection. In the following sections, we review DL-based techniques that have been applied so far for mono- and multi-omics analysis in brain cancer research. It discusses the application of DL techniques in the treatment of brain cancer from multiple perspectives. We start by describing DL architectures used in brain cancer research. After that, we discuss DL models developed based on single-omics datasets for predicting brain cancer. The review then shifts focus to discussing how the applicability of DL models that combine multi-omics data can enhance the precision of predictions. In addition, we discuss the application of DL models in the prediction of drug responses for the treatments of brain cancers. We conclude by discussing how one should construct DL frameworks for personalized medicine and the challenges in translating such models into clinical diagnostics.

Because the literature on DL with omics data specifically in brain cancer remains more limited than the broader cancer-omics literature, this review includes two categories of studies: (i) models developed or evaluated directly in brain tumor cohorts, including glioblastoma, lower-grade glioma, and pediatric brain tumors; and (ii) pan-cancer models that were not designed exclusively for neuro-oncology but provide methodological frameworks potentially transferable to brain cancer research. Throughout the review, we distinguish between these categories to avoid implying that pan-cancer performance necessarily reflects validated performance in brain tumor datasets. To align the review structure with this broad omics landscape, we discuss not only commonly used genomic, transcriptomic, miRNA, copy number alteration (CNA), and DNA methylation datasets but also briefly summarize emerging AI applications in proteomics and metabolomics, which remain less developed in neuro-oncology but are increasingly relevant for functional interpretation and treatment-response modeling.

Furthermore, the literature reviewed in this article was obtained by searching PubMed, Web of Science, Scopus and Google Scholar using keywords of brain cancer, glioblastoma, deep learning, artificial intelligence, genomics, transcriptomics, DNA methylation, miRNA, copy number alterations, proteomics, metabolomics, multi-omics, survival prediction, and precision medicine to provide an overview of the recent advances. Peer reviewed studies showing deep learning applications in brain cancer were given priority. Representative pan-cancer studies were included when computational frameworks were directly relevant to neuro-oncology.

## 2 Deep learning architecture utilized in the diagnosis and treatment of brain cancer

DL, a subdiscipline of machine learning, has revolutionized the identification and therapy of brain tumors through the ability to analyze intricate medical information to an unprecedented degree of precision. The older ML methodologies are founded upon manually selected characteristics such as the shape, size, or genetic makeup of the cancer. But DL dispenses with this through the ability to automatically learn intricate patterns from unprocessed information. This occurs through artificial neural networks, specifically deep neural networks, composed of various layers of linked nodes that take on higher-level traits from input information.

In the instance of brain cancer, DL models are able to process large volumes of imaging, genomics, and clinical data to support diagnosis and therapy. As an example, convolutional neural networks (CNNs) are used to scan through MRI scans to identify malignant tumors with an accuracy that is comparable to if not superior to that of human radiologists. The same can be accomplished using recurrent neural networks (RNNs) and transformer models to scan through patient histories and genetic profiles to predict outcomes. Using DL, researchers and clinicians are empowered to enhance early detection, personalize therapy options, and ultimately enhance patient survival.

DL models are crucial to modern healthcare, particularly medical imaging, genomics, and personalized treatment. The models are divided into three learning paradigms: supervised learning, unsupervised learning, and reinforcement learning. Supervised learning relies on labelled data and is most appropriate to tasks such as disease classification, cancer identification, and outcomes prediction. Unsupervised learning does not employ labelled data but is employed for pattern identification, feature extraction, and anomaly identification that are particularly useful in genomics (Alharbi and Vakanski, 2023). Reinforcement learning is used less in medical diagnosis but is most appropriate to sequential decision-making tasks such as the optimization of treatment plans or optimization of drug dosages based on patient response (Alom et al., 2019).

Supervised DL models include the feedforward neural networks (FNNs), or multi-layer perceptrons (MLPs), upon which most predictive models are based (Emmert-Streib et al., 2020). The networks use structured numeric information such as patient histories, genetic biomarkers, and laboratory test results to classify disease states or to predict treatment outcomes. While simple to create and computationally efficient, however, they are poor at extracting complex spatial or temporal relationships within medical data and are thus not very helpful for the analysis of imagery or time-series prediction.

Visual tasks are currently dominated by CNNs, particularly for medical imaging tasks like the detection of tumors from MRI and CT scans. CNNs employ convolutional layers to learn the spatial features from the images with minimum need for manual feature engineering. The learned features are then enhanced by the pooling layers to allow the high discriminative power of the CNNs to discriminate between cancerous and benign tumors. However, large annotated datasets and large computational resources are required to employ the use of CNNs, making them not appropriate for clinical use (Yamashita et al., 2018). For research into brain cancer, CNNs are employed extensively for classification, segmentation, and survival prediction purposes, sometimes integrating them with genomics information for a more comprehensive analysis. For genomic data, 1D-CNNs are often used for cancer classification, while 2D-CNNs are applied to image data (Mostavi et al., 2020). Various CNN architectures, including 1D-CNN, 2D Vanilla CNN, and 2D Hybrid CNN, have been employed to distinguish between tumor and normal samples using unstructured gene expression data from The Cancer Genome Atlas (TCGA). Shirazi et al., for example, successfully integrated image and genomic data to predict brain cancer patient survival using CNNs (Zadeh Shirazi et al., 2021). Besides diagnosis, CNN models have also been used to explore molecular mechanisms underlying gene expression, offering valuable insights into cancer biology.

RNNs are especially suited to sequential data such as patient medical histories and longitudinal imaging scans. While CNNs deal with spatial relationships, RNNs deal with temporal relationships and are therefore suited to longitudinally forecasting disease progression and response to treatment. Standard RNNs suffer from vanishing gradient issues that mean that they cannot learn to capture long-term dependencies. LSTM networks and GRUs are variations that address this issue by introducing specific gate mechanisms. RNNs are used in cancer research to analyze time-series genomics data to predict how cancer evolves under treatment (Wang et al., 2023a).

Unsupervised learning models that require no labeled data are particularly beneficial for the analysis of high-dimensional complex biological data. Restricted Boltzmann Machines (RBMs) are employed to discover hidden structures within genomics data, for example, to discover probable gene-disease relationships (Vrábel et al., 2020). Although giving valuable information about molecular mechanisms, RBMs are computationally costly and are difficult to optimize. Autoencoders are another form of unsupervised learning that are commonly employed for feature extraction and dimensionality reduction. Compressing the input data to a lower-dimensional space and then mapping it back to the input space enables autoencoders to highlight key biological characteristics relevant to cancer classification. This technique has gained widespread usage within the domains of transcriptomics and methylomics to aid the discovery of biomarkers for brain cancer (Doncevic and Hermann, 2023; Munquad and Das, 2023).

Generative Adversarial Networks (GANs) are being touted for the ability to generate artificial medical data to offset the limitation of having limited annotated datasets. The generator generates artificial data and the discriminator determines how real it is. In medical imaging, the use of GANs is to create real-looking artificial MRI scans to expand training sets for DL models. This technique is particularly useful for the identification of cancer in the brain where cancerous images are limited. But training the GANs is notoriously difficult and can generate artifacts if not carefully tuned. RBMs, which use unlabeled data to create nonlinear generative models, have been employed to predict miRNA-disease associations (Li et al., 2021; Chen et al., 2015). GANs and Autoencoders, more recent unsupervised approaches, do not require precise labeling for classification tasks (Ghahramani et al., 2018). GANs rely on a generative model that identifies patterns in input data to produce outputs, which has shown potential for improving classification accuracy across various domains, including genomics (Wei et al., 2022; Xiao et al., 2021). Autoencoders, which are feed-forward neural networks, reconstruct input data and have been widely used in cancer genomics, particularly for analyzing transcriptomic, methylomic, and mutational data (Franco et al., 2021; Qiu et al., 2020; Simidjievski et al., 2019).

Reinforcement learning is another form of DL that is directed towards sequential decision-making. In medicine, reinforcement learning is used primarily to optimize treatment strategies, such as dynamically adjusting chemotherapy doses based on patient response. The model learns by trial and error to maximize long-term patient outcomes. While there has been some evidence that reinforcement learning has proven to be beneficial to use for treatment planning, its reliance on simulated environments makes clinical validation problematic. In the treatment of cancer of the brain, models using reinforcement learning are under investigation to customize radiation therapy schedules to maximize the control of the cancer with the minimum possible adverse effect on normal tissue (Liu et al., 2022; Ribba, 2023). LSTM-based approaches include models like DanQ and DeepCare, the latter being an end-to-end deep dynamic network specifically designed to analyze electronic health records (EHR) for inferring current health conditions and forecasting future medical outcomes (Quang and Xie, 2016; Pham et al., 2016). Similarly, GRU networks have been utilized for the diagnosis and classification of cancer (Saboor et al., 2024).

Both of these DL methodologies are strong where the other is weak. The prediction and classification tasks are well established using the supervised learning methodologies such as RNNs and CNNs. The unsupervised methodologies such as autoencoders and GANs are essential for feature extraction and data augmentation. Reinforcement learning, in its early days in healthcare, has the capability to optimize intricate treatment decisions. The integration of these models, typically within hybrid models, is opening the doors to more accurate and personalized approaches to the diagnosis and treatment of cancer of the brain.

## 3 Deep learning and mono-omics data for brain cancer classification: current evidence and potential clinical utility

DL has emerged as a powerful tool in the classification of brain cancer using mono-omics data, offering significant advancements in clinical diagnosis. Mono-omics refers to the analysis of a single layer of biological information, such as genomics, transcriptomics, or proteomics. In brain cancer, mono-omics data can provide critical insights into tumor-specific molecular alterations (Malone et al., 2020). By applying DL algorithms to this data, complex patterns and associations that may elude traditional statistical methods can be uncovered, enabling accurate tumor classification and subtype differentiation (Yaqoob et al., 2023). This is particularly useful in identifying malignancy, assessing prognosis, and informing treatment strategies.

The application of DL in mono-omics data allows for enhanced pattern recognition by learning from large datasets, even when data is noisy or incomplete (Martorell-Marugán et al., 2019). For instance, DL models trained on gene expression profiles can distinguish between normal and cancerous tissues, predict tumor grade, and classify various brain cancer types, such as glioblastoma and astrocytoma, with high accuracy (Almars et al., 2021; Al-Azani et al., 2024; Joshi and Aziz, 2023; Steyaert et al., 2023; S Tandel et al., 2019). When validated with independent clinical datasets, DL-based models have demonstrated robust performance in brain cancer diagnosis, outperforming conventional ML techniques (Ahamed et al., 2023; Shoaib et al., 2025; Akinyelu et al., 2022). This integration of DL with mono-omics data not only supports personalized medicine but also holds promise for being translated into clinical settings as a non-invasive diagnostic tool. As the field advances, the exploration of multi-omics data in combination with DL offers even greater potential for enhancing cancer diagnosis and treatment. However, most DL models remain at the research or preclinical validation stage. Nevertheless, translation into routine clinical diagnosis requires independent external validation, model interpretability, and regulatory evaluation.

### 3.1 Gene expression data

Gene expression data is a foundational layer in the understanding of cancer biology, offering a genome-wide snapshot of transcriptional activity within cells. It is primarily generated through two high-throughput platforms: RNA-sequencing (RNA-seq) and microarrays. Because cancer is a complex, multifactorial disease involving various molecular alterations, capturing transcriptome-wide gene expression patterns becomes critical for understanding its initiation, progression, and response to therapy. Changes in gene expression reflect functional consequences of genetic and epigenetic aberrations that contribute to oncogenesis. Therefore, analyzing global gene expression can facilitate the identification of oncogenes and tumor suppressor genes, assist in biomarker discovery, and improve diagnostic and prognostic accuracy.

DL approaches have increasingly been applied to this domain due to their strength in modeling nonlinear and high-dimensional data. As shown in Table 1, numerous AI models have been developed to support precision oncology in brain cancer through analysis of gene expression data. One notable approach is DeepDCancer (Yuan et al., 2020), a two-component DL framework proposed by Yuan et al. for transcriptomic analysis. It combines an unsupervised deep autoencoder, DeepT2Vec, for dimensionality reduction and feature extraction, with a supervised classifier, DeepC, for cancer classification. DeepT2Vec compresses high-dimensional RNA-seq data into a latent representation using a five-layer autoencoder architecture. These latent features are then fed into DeepC, which performs classification of tissue samples as either normal or malignant. This hybrid framework integrates the strengths of unsupervised learning in discovering complex patterns and the accuracy of supervised learning in diagnostic prediction. DeepT2Vec/DeepC is likely a more robust architecture because it utilizes larger transcriptomic collections and unsupervised representation learning strategies to improve feature extraction. DeepDCancer demonstrated strong classification performance (Table 1) through unsupervised latent feature extraction, but its biological interpretability remains limited because latent embeddings do not directly correspond to clinically actionable pathways. The model was primarily evaluated on retrospective transcriptomic datasets, therefore, prospective validation across independent clinical cohorts is necessary before translational implementation.

**TABLE 1:** Summary of AI models were developed using transcriptomic or gene-expression data (Note: Single values indicate overall model performance, whereas multiple values correspond to different tumor types, subtypes, or classification tasks).

Model/Framework	Type of data used	Purpose	Methodology/Architecture	Advantages	Key results
DeepDCancer (Yuan et al., 2020)	RNA-seq data	Cancer diagnosis (pan-cancer, including GBM)	Deep autoencoder (DeepT2Vec) + Supervised classifier (DeepC)	Combines unsupervised feature extraction with supervised classification	Accuracy: 90% (pan-cancer), 94% (tissue-specific)
LS-CNN (Shah et al., 2020)	Gene expression microarrays	Cancer classification (pan-cancer, including pediatric medulloblastoma)	Laplacian Score-based gene selection + CNN	Handles high dimensionality; robust feature selection	Average Accuracy: 97%
CFNForest (Xu J et al., 2019)	Gene expression	GBM subtype classification	Deep Neural Network + Cascade of Decision Trees	Balances interpretability and flexibility	Accuracy: 85%
DeepGx (De Guia et al., 2019)	Gene expression	Cancer classification (pan-cancer including GBM and LGG classification)	Deep Neural Network	High scalability for large datasets	Accuracy: 95% (pan-cancer), 94% (GBM), 98% (LGG)
BCDForest (Guo et al., 2018)	Gene expression	GBM subtype classification	Boosted Decision Tree Ensembles	Greater transparency; reduced overfitting	Accuracy: 80.6%
DEcode (Tasaki et al., 2020)	Transcriptomic data	Gene expression prediction (pan-cancer, including GBM)	Multilayer Neural Network modeling transcriptional regulators	Captures complex gene regulation dependencies, improved interpretability	Accuracy: 78%

Another approach focusing on gene expression is the LS-CNN (Shah et al., 2020) model, developed by Shah et al., which integrates gene selection using the Laplacian Score with Convolutional Neural Networks (CNNs). The method begins with normalization and preprocessing of gene expression microarray data, followed by a hybrid feature selection process that combines filter-based statistical methods and wrapper-based optimization algorithms to identify the most informative genes. These selected features are input into a CNN architecture for classification, which employs nonlinear activation functions and a softmax output for multiclass prediction. This method achieved an average accuracy of 97%, outperforming conventional ML approaches in handling high-dimensional gene expression data. The LS-CNN framework highlights the importance of integrating feature-selection strategies with DL architectures for high-dimensional transcriptomic datasets. However, the authors noted that the high dimensionality and small sample size of the datasets utilized in the LS-CNN framework can raise the possibility of overfitting and potentially overestimate classification performance. To address the heterogeneity and high dimensionality of tumor data of glioblastoma (GBM), several DL-based tools have been developed to classify molecular subtypes from gene expression data. The CFNForest (Xu J et al., 2019) model combines a deep neural network for feature extraction with a cascade of decision trees, promoting both flexibility and robustness. In contrast, DeepGx (De Guia et al., 2019) relies solely on deep neural networks for subtype classification, optimizing for scalability and performance in high-dimensional spaces but with less interpretability. DeepGx converted RNA-seq expression profiles into two-dimensional image representations, which are subsequently classified *via* a CNN architecture. In a broad pan-cancer evaluation across 33 TCGA cancer types, DeepGx achieved an accuracy of approximately 95%; however, when applied specifically to the molecular subtyping of glioblastoma, the reported accuracy was 94%. A critical limitation of this approach is its heavy reliance on internal 10-fold cross-validation. Without independent external cohort validation, the model’s ability to generalize across the inherent biological noise of diverse, real-world patient populations remains unproven. BCDForest (Guo et al., 2018), meanwhile, employs a cascade of boosted decision tree ensembles that provide greater transparency and layer-wise training to prevent overfitting. While BCDForest emphasizes interpretability (achieving 80.6% accuracy), CFNForest offers a flexible compromise (85% accuracy), and DeepGx provides strong scalability with the highest accuracy (94%). The BCDForest model suffers from limited sample sizes, high dimensionality, and class imbalance, which can negatively impact classifier robustness. Considering CFNForest, DeepGx, and BCDForest together reveals a clear trade-off between predictive accuracy and interpretability. DeepGx scaled better and classified more accurately due to its deep neural representations, but it lacked biological transparency. BCDForest took the opposite approach, using boosted decision ensembles to favor interpretability over raw accuracy. Operating between these two, CFNForest attempted to balance feature flexibility with model explainability. These variations show that choosing a model requires looking beyond performance metrics to consider the specific clinical setting and the requirement of biological clarity.

Another relevant model is Decode, proposed by Tasaki et al. (2020), which aims to uncover the regulatory mechanisms underlying differential gene expression. It employs a multilayer neural network trained on large-scale transcriptomic datasets to learn interactions between transcriptional regulators, such as transcription factors and chromatin accessibility markers. This architecture captures non-linear, hierarchical dependencies within the gene regulatory landscape, offering interpretability regarding gene expression changes across biological conditions.

In parallel, DL has also been applied to infer gene expression from histone modification data. Tools like DeepDiff (Sekhon et al., 2018) and DeepChrome (Singh et al., 2016) serve this purpose. DeepDiff uses CNN layers to process ChIP-seq signals from histone modifications, followed by dense layers to predict whether genes are upregulated, downregulated, or unchanged. It captures the epigenetic context influencing transcriptional regulation and improves our understanding of chromatin dynamics. DeepChrome adopts a similar CNN-based architecture to predict gene expression from combinatorial histone modification patterns, further supporting the use of epigenomic features in modeling transcription.

Unsupervised DL techniques have also been used to cluster brain cancer samples based on gene expression profiles, which not only enhance classification accuracy but also help uncover novel cancer subtypes (Suo et al., 2019). These DL models enable more precise stratification of brain tumors and have significant implications for personalized medicine. Taken together, these gene expression based deep learning models handle high dimensional transcriptomic data well through feature extraction and dimensionality reduction. CNNs and deep networks usually deliver higher predictive accuracy, while ensemble and feature selection methods provide better interpretability and robustness. A common limitation, however, is that most studies relied on small retrospective cohorts, which raises valid concerns about overfitting, reproducibility, and how well these models generalize clinically. Table 1 summarizes the AI models and frameworks, which were developed using gene expression or transcriptomics data for pan-cancer and brain cancer for precision therapy.

### 3.2 Mutational profiles

Cancer lethality is intricately influenced by a combination of mutated genes, with numerous mutations collectively contributing to the remarkable heterogeneity and biological complexity observed within tumors, even among patients diagnosed with the same type of cancer. This heterogeneity has made the development of effective and universally applicable treatment strategies exceptionally challenging (Stratton et al., 2009). Mutations in cancer can broadly be categorized into two groups: driver mutations and passenger mutations. Driver mutations actively cause uncontrolled cell proliferation and play a direct role in oncogenesis, typically through structural changes in proteins that disrupt normal biological pathways, particularly when these mutations occur in tumor suppressor genes or oncogenes. In contrast, passenger mutations are largely biologically neutral and do not contribute significantly to cancer development. Nevertheless, the presence and accumulation of passenger mutations can still inform the evolutionary trajectory and heterogeneity of tumors (Munquad and Das, 2024; Arnedo-Pac et al., 2019).

The advent of high-throughput sequencing technologies has led to the generation of massive amounts of tumor genomic data, enabling researchers to combine gene mutation profiles with phenotypic information from cancer patients. However, despite these advances, how specific mutations translate into clinical outcomes remains poorly understood, which continues to impede the full realization of genomic medicine. Bridging this gap has been a major area of focus, and artificial intelligence (AI) and ML approaches have emerged as valuable tools in this endeavor. These approaches have been successfully applied in diverse studies for purposes such as the identification of driver genes (Tokheim et al., 2016; Bailey et al., 2018), the development of novel drugs (Cheng et al., 2019), and the advancement of precision oncology frameworks (Azuaje, 2019). Yet, challenges such as data sparsity, limited sample sizes, and high-dimensional feature spaces continue to hinder the optimal classification and interpretation of mutation-related genomic data in cancer research.

In response to these challenges, several DL models have been developed to improve cancer classification based on mutation data (Table 2). One such model, DeepGene, was introduced by Yuan et al. as a somatic point mutation-based cancer classification method that employs DL to uncover critical correlations between combinatorial point mutations and various cancer types (Yuan et al., 2016). DeepGene operates through a structured three-phase workflow. In the preprocessing phase, two main methods are employed: clustered gene filtering (CGF), which filters genes based on their mutation frequency to prioritize informative features, and indexed sparsity reduction (ISR), which focuses on reducing the overwhelming sparsity inherent in mutation matrices by concentrating on non-zero elements. Following preprocessing, the data are passed through a fully connected deep neural network (DNN) classifier. The DNN architecture enables the model to capture complex, non-linear interactions between mutations that traditional linear models like support vector machines (SVMs) fail to exploit fully. By capturing such highly interconnected features, DeepGene demonstrates improved classification accuracy across different cancer types. In a large-scale pan-cancer classification task involving 3,122 TCGA samples across 12 distinct tumor types, the model achieved an accuracy of 65.5%. While this figure is lower than those typically seen in transcriptomic studies, it underscores the inherent difficulty of training models on mutation matrices alone. A primary concern regarding this framework is its lack of validation on independent external cohorts, which limits its proven generalizability across broader clinical populations. Furthermore, while DeepGene tackled data sparsity and feature redundancy, the study did not thoroughly examine the potential dangers of data leaking during preprocessing and feature selection.

**TABLE 2:** Summary of DL models using mutation and copy-number alteration data for brain cancer precision therapy (Note: Single values indicate overall model performance, whereas multiple values correspond to different tumor types, subtypes, or classification tasks).

Model/Framework	Type of data used	Purpose	Methodology/Architecture	Advantages	Key results
DeepGene (Yuan et al., 2016)	Somatic point mutations	Cancer classification (Pan-cancer including LGG)	Fully Connected DNN	Reduces sparsity; captures mutation patterns	Accuracy: 65.5%
DeepCues (Zeng et al., 2021)	Whole-exome sequencing (somatic + germline mutations)	Cancer driver gene identification (Pan-cancer including brain)	CNN	Integrates somatic and germline variants; biomarker discovery	Overall accuracy: 77.6%
GDL Model (Sun et al., 2019)	Mutational sequence data	Classification and early detection (Pan-cancer including GBM, LGG)	Fully Connected Layers + Softmax output	High accuracy; effective early detection	Overall accuracy: 94.70%; GBM: 97.81%; LGG: 99.01%
CNA_origin (Liang et al., 2020)	CNA profiles	Tissue of origin classification (Pan-cancer including GBM)	CNN + Autoencoder hybrid model	Strong dimensionality reduction; good classification	Accuracy: 83.81%
Gao’s Multi-label Classifier (Gao and Baudis, 2021)	CNA data	Predict cancer type and tissue of origin (Pan-cancer including brain)	Multi-label Neural Network	Flexible output; multi-task predictions	F1-score: 0.75–0.67

Another notable DL approach, DeepCues (Deep Learning for Disease Classification using Exome Sequencing), was introduced by Zeng et al. to tackle cancer classification using raw DNA sequencing data (Zeng et al., 2021). DeepCues applies convolutional neural networks (CNNs) for automated feature extraction directly from whole-exome sequencing data. Distinctively, it integrates analysis of both somatic mutations and germline variants, offering a more comprehensive view of the genetic landscape of tumors. The primary architectural benefit of DeepCues was its capacity to acquire hierarchical sequence-level representations directly from raw genomic sequences without the need for feature engineering. In their evaluation, DeepCues achieved an accuracy of 77.6% across seven cancer types using an independent test set comprising 4,174 samples from The Cancer Genome Atlas (TCGA). When analysis was restricted to germline-only data, the model still achieved an impressive accuracy of 73.9%, suggesting that germline variants alone carry substantial predictive power. However, the lack of independent external validation and the limitation to predefined COSMIC cancer genes may constrain model robustness and biological generalizability across wider genomic contexts. On the positive side, DeepCues facilitated the identification of both known and novel cancer-driver genes, illustrating its potential for both classification and biomarker discovery.

Complementing these efforts, another DL model based on a genome deep learning (GDL) framework has demonstrated outstanding performance in classifying specific types of brain tumors (Sun et al., 2019). In this method, sequencing data is first aligned with a reference genome to identify mutations, followed by reformatting of the mutation information into a representation compatible with the GDL framework. Special emphasis is placed on low-frequency variation sites through targeted filtering to capture rare but potentially important signals. During the training phase, the model utilizes four fully connected layers culminating in a softmax regression output for classification. The GDL framework demonstrated a tiered performance by maintaining a robust overall accuracy of 94.70% across a pan-cancer genome analysis, but its precision in neuro-oncology was even more pronounced, yielding exceptional accuracies of 97.81% for glioblastoma (GBM) and 99.01% for lower-grade glioma (LGG). However, a noted limitation of this approach is its inability to predict cancer stages, which restricts its immediate clinical applicability for staging and prognosis estimation. Additionally, the performance substantially declined in the mixture model used for multicancer discrimination, suggesting difficulty in learning highly heterogeneous mutational patterns across tumor types. Nevertheless, given its high accuracy, the GDL model holds great promise for the early detection of brain cancers, which could enable preventive care and improved patient management.

It is noteworthy that the application of DL in cancer genomics has extended beyond driver mutations alone. Recent studies have explored the predictive value of patterns in passenger mutations, particularly in brain cancers such as GBM, where DL techniques have been used to distinguish primary tumors from metastatic cancers (Zeng et al., 2021). These findings highlight the broader utility of mutation-based DL frameworks in characterizing tumor biology and improving diagnostic accuracy.

By focusing on CNN and hybrid architectures, mutation based frameworks like DeepGene, DeepCues, and GDL manage sparse genomic mutation data effectively. These models improve cancer classification because they learn sequence level mutation patterns directly from the genomic data itself. The main bottleneck, however, remains the shortage of diverse mutation datasets and a general lack of external validation, both of which hinder clinical translation and reproducibility.

### 3.3 Copy number alterations (CNAs)

CNAs represent another important class of genomic aberrations with profound implications for cancer biology. CNAs involve the duplication or deletion of segments of DNA, often spanning large genomic regions or even entire genes, thereby causing significant changes in gene dosage and downstream gene expression (Araujo et al., 2021). Advances in whole-genome sequencing (WGS) and targeted sequencing have facilitated the comprehensive detection of CNAs, and recent studies have revealed distinct CNA signatures associated with different cancer types, indicating their potential utility for tumor classification, prognostication, and the development of personalized therapies.

Mechanistically, CNAs exert their influence through a variety of biological processes, including gene breakage, alterations in gene dosage, positional effects that influence regulatory landscapes, and the formation of novel gene fusions, all of which can contribute to tumorigenesis and disease progression (Poduri et al., 2013). The clinical relevance of CNAs has become increasingly apparent, especially in guiding tumor-targeted therapies and prognostic assessments. However, despite their biological importance, the application of DL to CNA data remains relatively limited compared to the more extensively explored domain of somatic mutations.

Nevertheless, notable advances have been made (Table 2). In one study, ML algorithms analyzing CNA data from GBM achieved an area under the receiver operating characteristic curve (AUC) of 0.875 when distinguishing GBM from other tumor types (Ko and Brody, 2021), demonstrating the feasibility of utilizing CNA profiles for cancer classification. Further highlighting the clinical importance of CNA analysis, key markers for lower-grade gliomas (LGG), such as 1p/19q codeletion and IDH mutations, are critical for distinguishing oligodendrogliomas from astrocytic tumors. Gradient boosting machines, a popular ensemble learning method, have been effectively applied to predict these hallmark genetic alterations based on CNA data (Nuechterlein et al., 2021).

Efforts to predict the tissue of origin for metastatic cancers have also benefited from the integration of DL with CNA profiling. In one notable study, CNNs combined with autoencoders were used to process CNA data, achieving an average training accuracy of 83.81% and an independent test accuracy of 79% on GBM datasets (Liang et al., 2020). This hybrid architecture leveraged the feature extraction capabilities of CNNs alongside the dimensionality reduction power of autoencoders to efficiently manage the complex and high-dimensional nature of CNA datasets. The success of this approach underscores the potential of DL frameworks to unlock hidden patterns within CNA data that are relevant for clinical classification.

Further advancing the field, Gao et al. developed a multi-label neural network classifier designed to simultaneously predict the cancer type and organ of origin using CNA data (Gao and Baudis, 2021). This innovative approach highlights the adaptability of DL models in handling complex biological data, as multi-label classification allows the model to make multiple clinically meaningful predictions from a single input profile. Although such approaches are computationally intensive and require careful balancing of multi-label datasets to avoid bias, they represent a significant step forward in realizing the potential of CNA data for comprehensive cancer diagnostics. The CNA-based models, despite their promising prediction capabilities, encounter significant drawbacks such as high dimensionality compared to small sample sizes, class imbalance, and cross-platform heterogeneity between sequencing and array technologies.

Although DL applications in CNA analysis are still emerging, the successes achieved so far suggest that CNA-based classifiers hold significant promise for improving tumor identification, characterization, and precision therapeutic strategies in the future. Table 2 lists the AI (artificial Intelligence) frameworks that have utilized mutation and CNA data for cancer classification in brain tumor cohorts.

### 3.4 MicroRNA (miRNA)

MicroRNAs (miRNAs) are short, non-coding RNA molecules typically comprising approximately 22 nucleotides, and they play a central role in post-transcriptional gene expression regulation. To date, the miRBase database contains over 38,589 entries across various species. The database contains 1917 precursors and 2656 mature miRNAs specifically within the human genome in the latest release (v22.1) (www.mirbase.org). These molecules are crucial for a broad spectrum of biological processes, including development, cell differentiation, proliferation, and apoptosis. The deregulation of miRNA expression is associated with the manifestation of multiple disease phenotypes, with particular emphasis on their role in cancer pathogenesis. Given their central importance in gene regulatory networks, miRNA expression profiles have received significant attention in cancer research, where alterations in miRNA levels have been consistently observed across various tumors such as lung cancer, colon cancer, leukemia, and glioblastoma (Takamizawa et al., 2004; Monzo et al., 2008; Calin et al., 2002; Hermansen and Kristensen, 2013).

Building upon the preceding discussion of gene expression profiling and DL methods for predicting tumor grades, subtypes, and survival outcomes in brain cancer, recent investigations have highlighted the unique predictive power of miRNA expression profiles. Studies have demonstrated that miRNA profiles can classify different tumor types with greater accuracy than gene expression data alone, underscoring the critical regulatory role that miRNAs exert within oncogenic processes (Lu et al., 2005). ML algorithms have been actively employed to leverage miRNA datasets for biomarker discovery, some of which have been listed in Table 3. For instance, a support vector machine (SVM) was applied to detect tumor-specific miRNAs in glioblastoma, achieving an accuracy of 81% in differentiating glioblastoma samples from healthy controls (Roth et al., 2011). This finding illustrates the potential of miRNA-based classifiers in distinguishing malignant from non-malignant tissues.

**TABLE 3:** Summary of DL models using miRNA expression and DNA methylation data for precision therapy.

Model/Framework	Type of data used	Purpose	Methodology/Architecture	Advantages	Key results
SVM on miRNA (Roth et al., 2011)	miRNA expression	Tumor detection (GBM)	SVM	Simple; effective on small datasets	Accuracy: 81%
CNN on miRNA (Bagheri Khoulenjani et al., 2020)	miRNA expression	Tumor detection (Pan-cancer including LGG)	CNN	Automated feature extraction; high accuracy	Up to 100% classification accuracy
deepTarget (Lee, 2016)	miRNA and mRNA sequences	Predict miRNA-mRNA binding	RNNs (LSTM, GRU) + Fully Connected Layers	No manual feature engineering; captures sequential dependencies	Accuracy: 96.41%
MethylNet (Levy et al., 2020)	DNA methylation profiles	Cancer Subtype prediction and feature interpretation (Pan-cancer including GBM and LGG)	Deep Neural Network + SHAP analysis	Automated preprocessing; scalable and interpretable	Accuracy: 97%
MRCNN (Tian et al., 2019)	DNA fragment methylation matrices	Cancer classification (Pan-cancer including brain cancer)	CNN + RNN hybrid	Captures both local and sequential methylation patterns	Accuracy: 93.2%
DNN + Autoencoder (Khwaja et al., 2018)	CpG island methylation data	Feature learning for classification (Pan-cancer)	Deep Autoencoder	Dimensionality reduction without manual intervention, cancer classification	Accuracy: 84.75%

(Single values indicate overall model performance, whereas multiple values correspond to different tumor types, subtypes, or classification tasks).

In parallel, the use of DL approaches, particularly CNNs, has significantly advanced the classification of cancers based on miRNA profiles. CNN-based models have achieved remarkable prediction accuracies, reaching 100% in some instances (Bagheri Khoulenjani et al., 2020), thereby demonstrating the exceptional capability of DL architectures to capture complex, high-dimensional patterns inherent in miRNA datasets. These developments indicate that miRNA signatures are not only potent indicators of tumor presence but also highly discriminative across different cancer types.

Among the most prominent advancements is the development of deepTarget (Lee, 2016), an innovative end-to-end deep learning framework specifically designed for miRNA target prediction. Accurate prediction of miRNA–mRNA interactions is fundamental for elucidating cancer regulatory mechanisms, biomarker discovery, and therapeutic target identification. deepTarget utilizes deep recurrent neural networks (RNNs), including architectures such as long short-term memory (LSTM) networks and gated recurrent units (GRUs), to process both miRNA and mRNA sequences. By employing RNN layers, deepTarget is able to capture sequential dependencies and complex interactions between miRNA and mRNA sequences, extracting meaningful features necessary for the accurate prediction of miRNA-mRNA binding sites. The model produces a probability score for predicted interactions through a fully connected layer with a sigmoid activation function and is trained in a supervised manner using binary cross-entropy loss. Operating in an end-to-end fashion, deepTarget eliminates the need for manual feature engineering, providing an automated and highly accurate approach to studying miRNA functionality and gene regulation. Its hybrid nature, combining both unsupervised and supervised learning strategies, enables the framework to achieve outstanding predictive performance. Notably, deepTarget attained a prediction accuracy of 96.41%, significantly enhancing the reliability of miRNA-mRNA interaction predictions. Although deepTarget was not developed or validated using brain tumor cohorts, its ability to accurately identify miRNA–mRNA regulatory interactions makes it a valuable computational framework for investigating post-transcriptional regulatory mechanisms that are relevant to cancer biology, including glioma. Nevertheless, the model was evaluated on a general human miRNA–mRNA interaction dataset rather than brain cancer-specific or pan-cancer clinical cohorts, and therefore its reported performance should not be interpreted as evidence of brain tumor diagnosis or classification.

The enhanced capabilities demonstrated by these DL models suggest that systematic and integrative analyses of miRNA expression, when coupled with advanced ML and DL algorithms, can significantly advance the clinical classification of brain tumors. Incorporating miRNA profiles into predictive modeling frameworks holds great promise for improving diagnosis, staging, and personalized therapeutic strategies in neuro-oncology. However, translating this high-level predictive promise into a routine clinical environment remains a significant challenge due to the technical demands of the models themselves. Specifically, deep learning models for miRNA analysis, particularly deepTarget and related RNN based approaches, show how sequential learning methods can isolate miRNA–mRNA interactions and regulatory signatures in brain cancer. While these architectures improve prediction sensitivity, they depend heavily on large training datasets. This dependency, combined with their computational complexity, often limits their scalability and routine use in clinical settings.

### 3.5 DNA methylation

DNA methylation is a crucial biochemical process where methyl groups are attached to the DNA molecule without altering its sequence, yet it exerts profound influence over gene expression. When methylation occurs at the promoter regions of genes, it often leads to hypermethylation, which typically suppresses transcription and results in decreased gene expression levels (Mallik et al., 2021). Conversely, a reduction in methylation, referred to as hypomethylation, is associated with enhanced gene expression. The aberrant regulation of these processes plays a significant role in cancer development, where hypermethylation of tumor-suppressor genes and hypomethylation of oncogenes are commonly observed (Baylin and Herman, 2000; Ehrlich, 2009). As a result, the methylation status of promoter regions has emerged as a valuable biomarker for cancer diagnosis. Beyond gene expression regulation, DNA methylation also participates in fundamental biological processes such as X-chromosome inactivation and genomic imprinting, making the examination of methylation patterns critical for understanding disease progression. In view of its central role across multiple cellular functions, methylome data has become increasingly important for cancer classification and diagnosis, leading to a surge in studies applying DL techniques to analyze DNA methylation profiles (Jin et al., 2011).

Several cutting-edge DL frameworks have been developed to identify DNA methylation indicators for diagnosing various cancer types (Table 3). One such example is MethylNet, a user-friendly DL framework specifically designed for the analysis of DNA methylation (DNAm) data (Levy et al., 2020). MethylNet supports a range of tasks, including unsupervised embedding, supervised classification, and multi-output regression. It adeptly compresses high-dimensional data into lower-dimensional spaces, enabling clustering even when labelled DNAm datasets are unavailable. This automated and modular platform uses a deep neural network (DNN) with multiple hidden layers, non-linear activation functions such as ReLU, and dropout regularization to model complex relationships within methylation data. MethylNet streamlines preprocessing steps like dimensionality reduction and feature selection and allows customizable outputs, including class probabilities *via* softmax for classification tasks or continuous values for regression. Moreover, the model incorporates interpretability features through the application of SHAP (SHapley Additive exPlanation) values (Lundberg and Lee, 2017), a model-agnostic interpretative method based on cooperative game theory. SHAP elucidates individual model predictions by attributing feature contributions, ensuring local accuracy and providing intuitive explanations. Through its intuitive command-line interface and automation capabilities, MethylNet offers a scalable and efficient toolset that facilitates research in epigenetics and the clinical application of DNA methylation data.

Another important advancement is Methylation Regression Convolutional Neural Network (MRCNN), which integrates CNNs and RNNs to analyze methylation data (Tian et al., 2019). MRCNN begins with a matrix input representing a DNA fragment centered on a methylation site, utilizing one-hot encoding to capture nucleotide information. It reshapes this data into a two-dimensional tensor, enabling CNN layers to extract local sequence patterns while RNN components, particularly designed for capturing long-range dependencies, learn sequential relationships. This layered architecture progresses through convolutional and pooling operations, with further convolutional layers correcting for saturation effects before the model’s final fully connected layer generates the predicted values. MRCNN thus offers a sophisticated approach to capturing both spatial and sequential features of DNA methylation data.

A separate DL framework has been developed to predict and classify DNA methylation patterns by distinguishing healthy datasets from cancerous ones (Yassi et al., 2023). This system integrates an algorithm capable of identifying and imputing missing CpG island states, followed by precise mathematical feature extraction methods to isolate critical distinguishing characteristics. By combining this approach with a deep autoencoder, the model autonomously learns to classify cancer types (Khwaja et al., 2018). The deep autoencoder serves a dual purpose, simultaneously performing dimensionality reduction and feature learning, which is particularly crucial when handling the high-dimensional nature of methylome data.

DeepCpG, another DL-based method, focuses on predicting methylation states and detecting changes in methylation levels (Angermueller et al., 2017). Unlike traditional approaches that separate feature extraction from model training, DeepCpG employs a modular architecture that simultaneously learns from both DNA sequences and methylation patterns. The model is composed of three key components: the DNA module, the CpG module, and the Joint module. The DNA module employs convolutional and pooling layers to discover predictive motifs in local sequences, followed by fully connected layers modeling motif interactions. The CpG module, incorporating a GRU network operating bidirectionally, examines the neighborhood context around CpG sites within cells. The Joint module then integrates features from both the DNA and CpG modules, generating higher-level representations that enable precise methylation state predictions across individual cells. By modeling the interactions of adjacent CpG sites within and between cells, DeepCpG offers a powerful framework for genome-wide investigations, helping identify missing CpG sites and uncover sequence patterns associated with methylation variability. However, despite these advantages, DeepCpG was predominantly trained and tested on experimentally generated single-cell methylation datasets with very low CpG coverage and minimal sample, which could restrict its adaptability in highly variable clinical datasets.

MethPed represents another significant tool developed to classify pediatric brain tumors using DNA methylation patterns (Danielsson et al., 2015). MethPed applies a ML approach that utilizes methylation profiles at specific CpG sites as input features. It employs classification algorithms such as random forests or support vector machines, trained on curated datasets of pediatric brain tumor samples, to reliably assign tumor subtypes based on methylation patterns. MethPed thus provides a clinically robust method for diagnosing and stratifying pediatric brain tumors, significantly aiding treatment planning. MethPed performed effectively (98.3%) in methylation-based tumor classification, although many methodological limitation should be considered. The model was verified on 18 cancers, but several tumor subclasses were underrepresented, which may cause class imbalance and impact generalizability. Even while Random Forest classifiers are resistant to overfitting, a huge number of methylation probes relative to sample size can still cause model bias and instability, especially for rare tumor types like ETMR (Embryonal Tumor with Multilayered Rosettes). MethPed’s multiclass Random Forest design allowed robust classification of heterogeneous pediatric brain tumor subtypes and identification of biologically meaningful methylation signatures associated with clinically relevant subclasses.

Working with methylome data is incredibly difficult because dealing with over 800,000 CpG sites creates a massive feature extraction bottleneck. When the number of probes vastly outweighs the sample size, it easily introduces model bias and instability, which is a problem that impacts almost any framework built on this data. Frameworks like MethylNet and DeepCpG try to handle this by using strong feature-learning capabilities to improve epigenetic profiling and tumor subtype prediction, sometimes even pairing them with explainable AI for better interpretability. But even with these tools, real-world deployment remains tough; cross-platform batch effects, sparse CpG coverage, and cohort heterogeneity constantly get in the way of reproducibility and external testing. A detailed breakdown of these and other AI models developed for miRNA or methylation data is provided in Table 3.

### 3.6 Proteomics data

Proteomic profiling has become an essential method for systematically identifying functional perspectives of any disease or tumor biology that is not fully understood from genomic, transcriptomic and epigenomics data. However, AI models, specifically DL models using only proteomics data in brain cancer, are not reported due to various biological and technical limitations. One of the primary challenges may be the instability and dynamic character of proteins since protein expression is very context-dependent and affected by post-translational modifications. This results in proteome profiles being less consistent across cohorts and platforms, limiting robust training of AI models. A study by (Wang et al., 2023b) demonstrated that cancer proteomic profiles exhibit distinct molecular patterns. In particular, proteome-based stratification of gliomas identified three proteomic subgroups that may supplement current WHO classifications and provide a basis for subtype-specific targeted therapy. Results further show that AI approaches may be increasingly employed in the future for proteome-based diagnostic analysis. Their utility, however, is going to rely on the development of proteomic technologies that can yield more robust and quantitative data.

### 3.7 Metabolomics data

Metabolomics is an emerging field that aims at measuring all metabolites and other small molecules in biological samples. Owing to its broad coverage of biological processes and metabolic pathways, metabolomics holds significant potential as an objective molecular framework for precision medicine. Complex diseases such as cancer are known to induce measurable, often tissue-specific, changes of metabolite profiles. Only a limited number of AI-based models have been developed for metabolomics data analysis, with the majority of studies relying primarily on ML approaches. However, several factors restrict their broader applicability, including small patient cohort sizes and the lack of independent external validation, both of which may compromise model robustness and generalizability.

Recently, the integration of metabolomic data with DL and ML frameworks have shown that it can considerably enhance the classification of tumors. For instance, in glioblastoma, plasma metabolomic and lipidomic profiles analyzed with gradient-boosting and random forest models achieved predictive accuracies of approximately 92% and 85.7%, respectively, in differentiating tumor stages and recurrence patterns, highlighting the translational potential of AI-assisted metabolomics in precision oncology (Hodeify, 2024; Aboubechara, 2025). Moreover, advanced machine-learning techniques, including EvoHDTree, support vector machines, and DL models, have demonstrated promising results in distinguishing LGG, high-grade glioma, and meningioma based on metabolite panels in plasma, with classification accuracies and F1-scores reaching up to 91%–98.5% (Godlewski et al., 2023). These AI-based metabolomic techniques promise to facilitate early diagnosis, individualized therapeutic decision-making, treatment response monitoring, and the discovery of metabolically susceptible pathways to further precision medicine for patients with glioma and LGG.

Mono-omics models provide valuable insights into individual molecular layers, but they are inherently limited to one dimension of a complex biological system. However, brain tumors are under the complicated regulations of genetics, epigenetics, proteomics and metabolism. Thus, the shift to multi-omics integration is not only a benefit, but a necessity for the development of models that reflect true tumor heterogeneity. The utilization of these data from various biological layers can improve the support for predictive frameworks for precision therapy. Therefore, in the next section, we have discussed the multi-omics model with deep leaning and their applications.

## 4 Integration of omics data for brain cancer classification

In previous sections, we explored the intricate, multilayered interactions within the genomic landscape, such as the way miRNA expression levels influence mRNA abundance, or how promoter region methylation regulates gene expression. However, analyzing these molecular layers independently does not capture the full complexity of cancer biology. The utilization of any single type of omics data alone is generally insufficient to accurately predict tumor development and progression. A deeper understanding of cancer requires integrating interaction across multiple genomic layers. Therefore, effective multi-omics integration strategies are essential, especially considering that multi-omics data often come from diverse and disparate sources (Menyhárt and Győrffy, 2021). This creates one of the central challenges in precision medicine: finding robust methods to cohesively integrate highly heterogeneous, disorganized datasets in order to improve diagnostic accuracy.

To address the complexity inherent in genomic and multi-omics data, researchers have increasingly adopted DL models, particularly autoencoders (Chai et al., 2021; Ballard et al., 2024; Koumakis, 2020). Autoencoders are an unsupervised learning technique well-suited for detecting meaningful patterns in large, heterogeneous datasets. Structurally, an autoencoder comprises an encoder, a bottleneck layer, and a decoder. The encoder compresses input data into a latent representation, while the bottleneck captures its most essential features in a reduced dimensional form. Subsequently, the decoder reconstructs the original input from this latent space. This compression and reconstruction process allows autoencoders to streamline complex genomic data into more manageable and informative representations, making them valuable tools for downstream ML applications in genomics (Li et al., 2022). By capturing the underlying structure of the data, autoencoders facilitate more accurate downstream analyses. In particular, they have been widely used to integrate multi-omics datasets for disease classification and the identification of cancer subtypes (Picard et al., 2021). Since accurately classifying cancer subtypes requires accounting for multiple biological layers, integrating various omics data types becomes critical.

In multi-omics analysis, two main strategies for data integration are commonly employed: early integration and late integration. Early integration involves merging all available omics data from a sample into a single dataset before applying feature learning methods and model training, enabling deep learning models to learn shared molecular representations across different biological layers. Conversely, late integration process learn features separately from each omics data type and then combines these learned representations for downstream prediction.

Autoencoders have shown considerable success in early integration, where latent representations learned from multiple omics datasets facilitate tasks like subtype classification and clustering, as demonstrated in glioblastoma studies (Madhumita and Paul, 2022). Wissel et al. (2022) proposed multiple neural network integration techniques, demonstrating that hierarchical autoencoder-based late integration of modality-specific data outperformed other state-of-the-art approaches in glioma analysis.

The DeepOmix framework represents an early integration strategy for multi-omics cancer, including glioma, for survival analysis (Zhao et al., 2021). It integrates gene-level data from different omics sources, such as gene expression, methylation, and mutation profiles, through a biologically informed five-layer neural network. The model begins with an input layer representing genes, which maps to a functional module layer corresponding to known biological pathways, enhancing the model’s interpretability. These pathway-level features pass through hidden layers that capture complex, non-linear interactions, ultimately outputting a survival risk score based on a Cox proportional hazards model. DeepOmix is not only scalable but also interpretable, providing accurate survival predictions alongside biologically meaningful insights. Benchmark evaluations have demonstrated that DeepOmix outperforms several advanced methods, including IPF-LASSO (Boulesteix et al., 2017), glmboost (Hofner et al., 2014), block forest (Hornung and Wright, 2019), DeepSurv (Katzman et al., 2018), and DeepHIT (Lee et al., 2018). The ability to uncover critical cellular processes and molecular interactions through multi-omics analysis will be vital in discovering novel diagnostic and therapeutic strategies for cancer treatment. Although DeepOmix introduced an interpretable multi-omics DL architecture, the model primarily relied on TCGA-derived cohorts, lacked extensive evaluation on independent external validation datasets, and did not report cross-platform reproducibility.

Recently, a DL model that integrates multi-omics data with a CNN-based early integration architecture has achieved highly accurate classification of glioblastoma subtypes using transcriptome and methylome data (Munquad et al., 2022). Balancing computational accuracy with biological interpretability, this model achieved an impressive classification accuracy of 98.20%, surpassing traditional ML approaches. It also demonstrated biological relevance by uncovering genotype-phenotype relationships and identifying subtype-specific biomarkers critical for diagnosis and treatment optimization.

DL techniques, particularly autoencoders, have also been successfully combined with other algorithms like XGBoost to identify cancer-associated genes (Mohammed et al., 2023). Furthermore, a multimodal DL tool called MultiSurv, has been developed to deliver accurate long-term survival predictions for cancer patients (Vale-Silva and Rohr, 2021). MultiSurv represent a late integration architecture comprises of multiple dedicated submodels, each designed to process a different data modality—including clinical information, mRNA, miRNA, DNA methylation, CNA data, and whole-slide pathology images. Each submodel generates a 512-dimensional feature vector, which are then fused through an element-wise maximum operation to form a unified patient representation. This consolidated vector feeds into a fully connected neural network that outputs conditional survival probabilities across 30 1-year intervals. MultiSurv does not assume proportional hazards, allowing it to offer flexible and precise long-term survival predictions across multiple cancer types, with the entire system trained end-to-end for optimal accuracy. From an architectural perspective, MultiSurv included numerous key advances including modality-specific submodels, multimodal feature fusion layers and discrete-time survival prediction which avoids the proportional hazards assumption of Cox-based models. MultiSurv demonstrated a good prognostic accuracy but lacked prospective external validation and analyses on clinical reproducibility.

Similarly, Zhang et al. (2021) developed OmiEmbed, a multi-view, multi-task DL late integration framework designed to integrate high-dimensional multi-omics datasets. Central to OmiEmbed is a deep embedding module based on a variational autoencoder (VAE) structure. Different types of omics data are compressed into a shared, low-dimensional latent space *via* individual encoders and decoders for each modality. This unified latent representation is then fed into several downstream task modules that handle classification, regression, and survival prediction simultaneously, leveraging multi-task learning to enhance generalization and predictive power. OmiEmbed undergoes a three-stage training process involving unsupervised pre-training of the embedding module, supervised pre-training of task modules, and final end-to-end fine-tuning to maximize its performance. The main architectural advantage of OmiEmbed resides in its multi-task learning method, in which a shared latent embedding serves many downstream clinical tasks simultaneously, enhancing the efficiency of representation learning, and eliminating task-specific redundancy. Moreover, the approach used deep embedding modules to reduce the “curse of dimensionality” of omics data with thousands of molecular features but relatively small patient cohorts. Therefore, the framework has shown potential to deliver highly precise, personalized treatment strategies for cancer patients. The AI-based approaches implemented in cancer precision therapy using different types of omics data are summarized in Table 4.

**TABLE 4:** Summary of multi-omics DL models developed for pan-cancer and brain cancer precision therapy (where C-index denotes the concordance index).

Model/Framework	Type of data used	Purpose	Methodology/Architecture	Advantages	Key results
DeepOmix (Zhao et al., 2021)	Multi-omics data	Survival prediction (Pan-cancer)	Biologically-informed Neural Network + Cox model	Enhances interpretability; captures non-linearities	High C-index, p < 0.0001
Block Forest (Hornung and Wright, 2019)	Multi-omics with block structures	Survival prediction (Pan-cancer)	Random forest variant tailored for grouped covariate structures	Preserves group structures (e.g., omics blocks); enhances interpretability	Mean C-index >0.6, adjusted p-value - 0.027
CNN-based Glioblastoma Classifier (Munquad et al., 2022)	RNA-seq + DNA methylation (GBM-specific)	Glioblastoma subtype prediction	CNN architecture combining transcriptome and methylome data	High accuracy with interpretability	Accuracy: 98.20%
MultiSurv (Vale-Silva and Rohr, 2021)	Multi-modal (clinical, mRNA, miRNA, methylation, CNA, histopathology images)	Survival prediction (Pan-cancer)	Deep multimodal fusion + survival modeling	No proportional hazards assumption; flexible survival modeling	Maximum C-index: 0.988
OmiEmbed (Zhang et al., 2021)	Multi-omics (mRNA, miRNA, methylation)	Diagnostic, survival prediction (Pan-cancer including brain tumor)	Variational Autoencoder (VAE)-based multi-task framework	Personalized predictions across multiple tasks	C-index: 0.77
MCMOCL (Mohammed et al., 2023)	Gene expression + methylation data	Cancer gene discovery (Pan-cancer)	Autoencoder for feature extraction + XGBoost	Improves feature selection; robust detection	Accuracy: 98%

Altogether, these findings illustrate how DL-based frameworks are becoming essential tools for integrating and analyzing multi-omics data, helping to revolutionize the diagnosis and treatment of brain cancer (Figure 2). AI proves exceptionally capable of handling high-dimensional, genome-wide datasets, uncovering hidden patterns that might be missed when analyzing isolated types of genetic data. By transforming vast datasets into clinically actionable insights, AI is accelerating the progress of precision medicine. Multi omics frameworks like DeepOmix, MultiSurv, and OmiEmbed push precision oncology forward by combining heterogeneous molecular and clinical data into single predictive systems. These models generally yield better prognostic and survival predictions than single omics approaches can manage. However, actually deploying them remains difficult. Issues with data harmonization, high computational complexity, and a lack of multicenter validation mean that despite their strong translational potential, these platforms are still largely restricted to preclinical settings.

**FIGURE 2:**
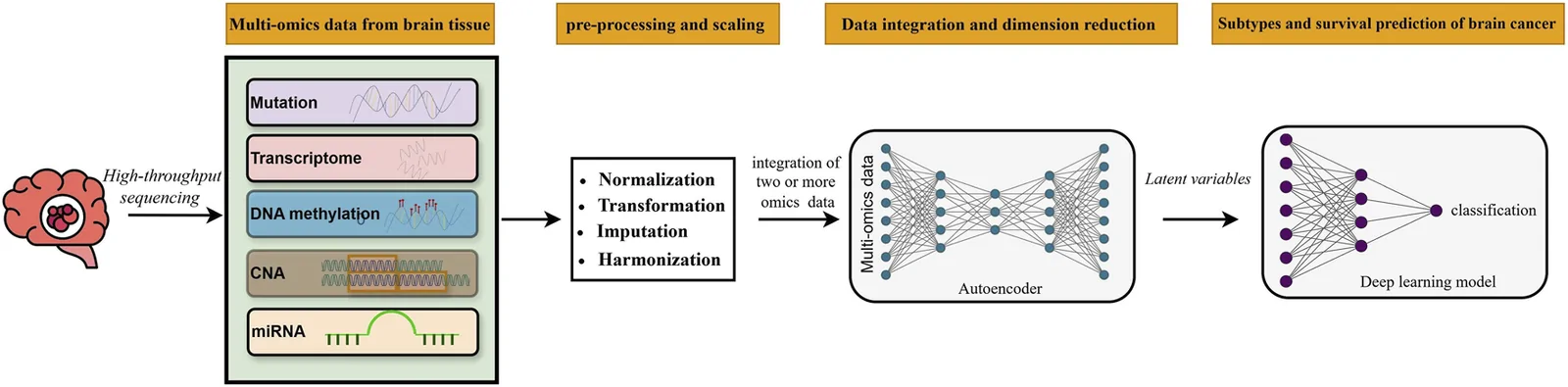
Integration of multi-omics data: Multi-omics data integration for brain cancer classification and survival prediction involves preprocessing the omics data in the initial step. Next, the data is integrated using an autoencoder, generating latent features that are subsequently utilized as input for a DL model to perform classifications, including subtype identification and survival prediction.

## 5 AI and multi-omics approaches in drug response prediction and personalized medicine

Precision medicine is a strategy aimed at developing personalized care for patients by considering their unique molecular profiles. This approach focuses on exploring the relationship between genomic alterations and their contribution to the risk of developing specific cancers, as well as their effects on treatment outcomes (Krzyszczyk et al., 2018). Different patients often exhibit varied responses to the same treatment due to intratumor heterogeneity, which can negatively impact survival rates despite the availability of numerous cancer therapies (Marusyk et al., 2020; Das et al., 2020).

A significant challenge in oncology research is accurately predicting individual responses to the treatments, leading to issues such as drug resistance (Zargar et al., 2020). Typically, oncologists rely on trial-and-error methods that can increase the side effects of anticancer drugs, damaging normal cells along with cancerous ones (Anand et al., 2022). To better understand the relationship between genomic profiles and drug sensitivity, DL methods have emerged as powerful tools. These techniques significantly enhance the capabilities of computer-assisted exploration in drug development, allowing for more precise predictions of treatment efficacy based on comprehensive multi-omics data (Johnson et al., 2021). By integrating various genomic layers, researchers can develop targeted therapies that minimize side effects and improve patient outcomes, ultimately advancing the field of precision medicine (Wang and Wang, 2023).

The advent of AI in drug discovery has garnered significant interest due to its potential to dramatically reduce both the time and cost associated with developing new pharmaceuticals (Marques et al., 2024). Two notable applications of DL in this context are *de novo* drug design and drug-target interaction (DTI) prediction. By leveraging multi-omics data, DL models can identify how molecular profiles influence cancer drug responses, thereby enhancing the relevance of precision medicine (Kim et al., 2021; Askr et al., 2023). These computational models for predicting drug sensitivity play a crucial role in selecting and prioritizing candidate compounds for pre-clinical studies. A general workflow for drug-response prediction using multi-omics data and AI-based integration strategies is shown in Figure 3.

**FIGURE 3:**
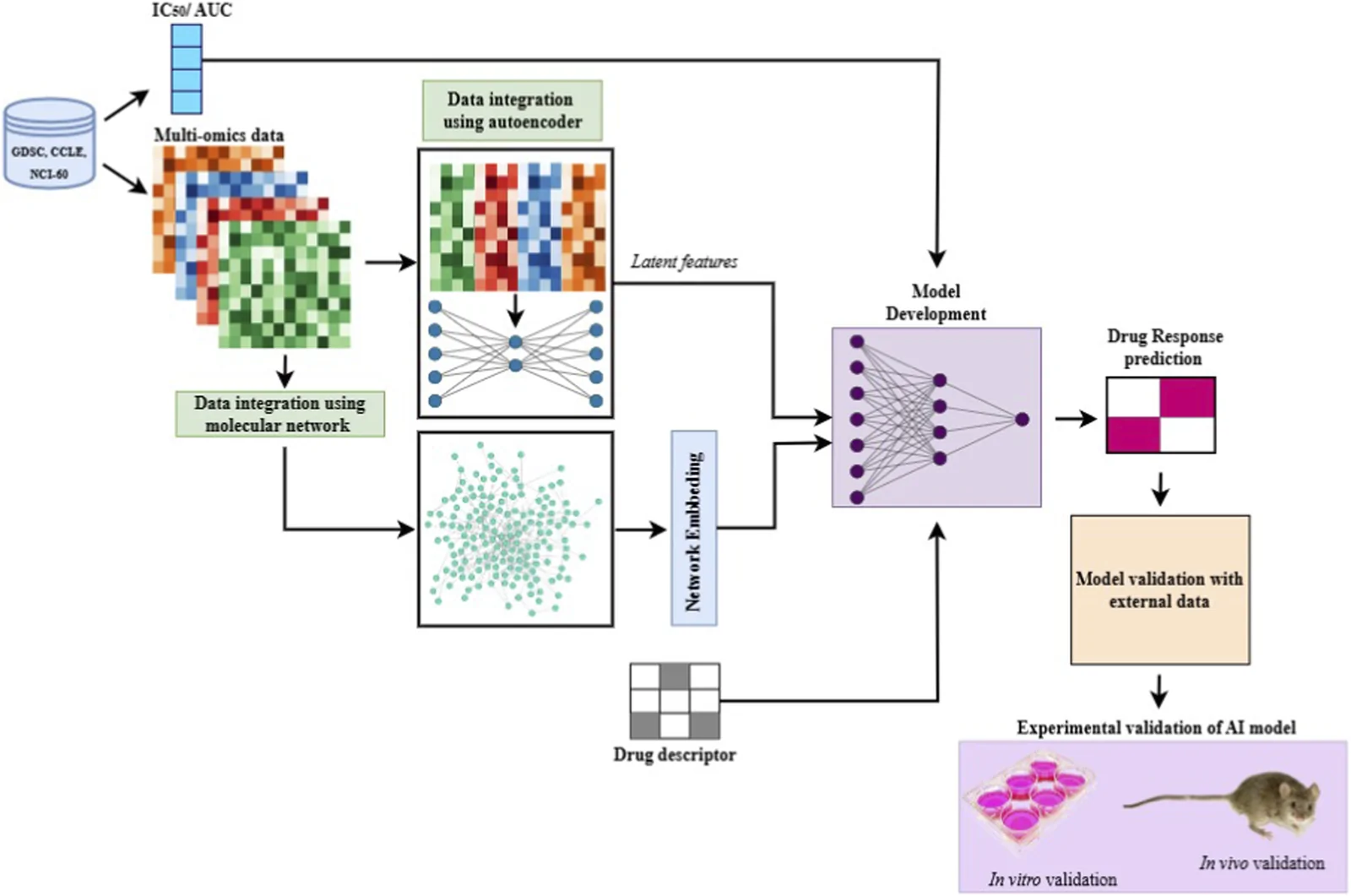
An overview of drug response prediction models utilizing multi-omics data and AI techniques. Multi-omics data can be integrated through two primary methods: autoencoder-based and network-based approaches. In the autoencoder-based method, data integration produces latent variables from the embedding space, which are subsequently used for predicting drug response. Conversely, the network-based approach integrates data *via* molecular networks, and the resulting embedded features are employed for model development.

One innovative approach, known as Cancer Drug Response profile scan (CDRscan), utilizes a convolutional neural network architecture to forecast anticancer drug responsiveness based on genomic profiles from 787 human cancer cell lines and 244 drugs (Chang et al., 2018). It consists of two parallel CNN “scan” modules—one that processes binary-encoded genomic mutation profiles and another that processes molecular fingerprints of drugs (e.g., from SMILES strings). These scan modules extract feature representations from each input, which are then fused into a combined feature vector. This fused representation is passed through fully connected layers to predict drug effectiveness, measured as IC50, capturing complex interactions between genetic features and drug structures. Impressively, the model gives very high prediction accuracy, with *R* ^2^ values higher than 0.84 and an area under the receiver operating characteristic curve (AUROC) of more than 0.98. Importantly, this is the first instance where a DL algorithm has been used to evaluate the feasibility of drug repurposing.

The statistical models, with the data of various cancer tissues and multi-omics profiles, had attempted earlier to predict drug response and identify biomarkers. It integrates diverse molecular data types—such as gene expression, CNA, and mutations—while modeling tissue-specific effects through varying coefficients, enabling the identification of both shared and cancer-type-specific biomarkers. The model also incorporates drug-drug similarity using a tree-structured multi-task learning penalty, improving prediction across related drugs. Furthermore, it effectively handles missing drug response values *via* a projection operator and optimizes the high-dimensional model using the smoothing proximal gradient method, offering both predictive accuracy and interpretability (Zhao et al., 2022). This model is capable of identifying tissue-specific biomarkers that can predict multi-drug responses.

Individualized drug response prediction is crucial to the forward-looking advances in precision medicine and the development of patient-tailored cancer therapies. Large-scale multi-omics profiles of characterization provide unprecedented opportunities for targeted cancer therapies by characterizing glioma pathways through the integration of multi-omics data within the context of a panel of gene expression and DNA methylation profiles of individual drug responses.

Additionally, a user-friendly web interface called CancerDAP was established by Xu et al. involves integrating multi-omics data (including gene expression, mutation, and methylation profiles) with drug response data to identify predictive subpathway signatures for individualized anticancer therapy to facilitate the exploration of 2,751 subpathways associated with 191 anticancer drug responses (Xu Y et al., 2019). Instead of analyzing entire pathways, the approach extracts smaller, functionally relevant subpathways using graph-based algorithms. These subpathways are then used to construct multi-layered feature sets for each sample, which are input into ML models (e.g., SVM, Random Forest) to predict drug sensitivity. The resulting subpathway signatures enable personalized drug response prediction and offer biological insights into treatment mechanisms.

F. Iorio et al. further involves integrating large-scale drug response data from cancer cell lines with comprehensive genomic profiles to uncover predictive biomarkers of drug sensitivity. The authors use Elastic Net regression models to link genomic features—such as mutations, CNAs, and gene expression—with responses to 265 anti-cancer drugs (Iorio et al., 2016). These models identify statistically robust associations, which are further interpreted at both the individual feature and pathway levels to uncover known and novel pharmacogenomic interactions. The resulting landscape serves as a resource for precision oncology, offering insights into drug mechanisms, resistance, and potential therapeutic strategies. Their research highlighted how these alterations are associated with specific data types in tumors and their relationships with drug sensitivity and resistance.

Inter- and intra-tumor heterogeneity contribute to the genotypic differences observed among patients, underscoring the necessity of personalized medicine for effective treatment. With the vast amounts of available data and high-throughput experimental techniques, DL has the potential to transform decision support systems in oncology, revealing hidden phenotypic and genotypic patterns along with their correlations (Bhinder et al., 2021; Dincer et al., 2018). The DeepProfile framework exemplifies this potential, utilizing unlabeled gene expression data to predict complex disease phenotypes (Dincer et al., 2018). DeepProfile can reveal complex, non-linear relationships between gene expression and phenotype data. This framework can be applied to brain cancer gene expression datasets in order to understand the relationship between genotype and phenotype which will help with the design of personalized treatment. The heterogeneity is an inter-tumor and intra-tumor factor contributing to variations in drug response. The architecture of the DeepProfile model is based on a deep autoencoder designed to learn low-dimensional representations of high-dimensional cancer gene expression data. It consists of an encoder that compresses gene expression profiles into a compact latent space, and a decoder that reconstructs the original input from this representation, trained using reconstruction loss (mean squared error). Once trained, the low-dimensional latent features serve as biologically meaningful embeddings that can be used for downstream predictive tasks such as drug response prediction and cancer subtype classification, enabling improved precision medicine applications. A deep variational autoencoder (VAE) model has been shown to efficiently predict drug responses by effectively handling these heterogeneous datasets (Jia et al., 2021). Furthermore, this approach enabled the identification of molecular characteristics linked to drug response across 33 cancer types, including brain cancer (Figure 3).

Establishing the relationship between genotype and phenotype is essential for uncovering the molecular signatures of diseases. GenNet is a DL framework developed to forecast phenotypic traits based on genetic variations. It uses SNPs as input, which are connected to genes through predefined biological mappings based on genomic annotations, and optionally to biological pathways, forming structured layers that reflect real biological relationships. Each connection in the network represents a known biological link, rather than being learned arbitrarily, enabling clear interpretation of the model’s predictions. The final output layer predicts the phenotype, making the entire architecture both transparent and biologically meaningful, while still leveraging the predictive power of DL (van Hilten et al., 2021). Additionally, Varmole, another biologically interpretable tool, integrates multi-omics network data within a deep neural network framework to prioritize variants, genes, and regulatory connections (Nguyen et al., 2021). This tool improves the prediction of genotype-phenotype relationships, deepening the understanding of underlying biology. Varmole is a gray box model with a biologically interpretable structure. Its architecture consists of an input layer of SNPs connected to a gene expression layer through biologically validated eQTL links, using a DropConnect strategy to randomly drop connections while preserving only known SNP-gene associations. This is followed by a gene-gene interaction layer informed by gene regulatory networks, allowing only biologically plausible gene interactions. The final output layer performs disease classification, and the structure of the model enables interpretable prioritization of variants and genes based on learned weights. It has four layers: (i) an input layer of SNPs and genes, (ii) a transparent layer mirroring input genes, (iii) hidden layers, and (iv) a phenotype output layer. The SNPs, genes, and transcription factors’ regulatory interaction are modelled by gene regulatory networks and expression quantitative trait loci.

To target therapy of complex conditions, for example, brain cancer, personalized medicine tailored to individual molecular profiles will be crucial. Precise predictions of the responses of medications are an essential step toward forwarding precision medicine and the construction of customized treatments for cancer. The integration of large-scale multi-omics data offers remarkable potential for precision therapy (Raufaste-Cazavieille et al., 2022). Moreover, employing a DL-based support system is highly desirable in contemporary medicine to mitigate drug-related side effects and enhance the life expectancy of brain cancer patients. The application of AI enables the synthesis of genomic information across multiple molecular layers, which can significantly enhance prognostic predictions regarding patient outcomes, including the likelihood of a favorable response to cancer treatment interventions (Liao et al., 2023; Partin et al., 2023).

The integration of multi-omics data, which encompasses diverse biomolecular information, holds significant potential for enhancing our understanding of intricate biological mechanisms. Argelaguet et al. (2018) introduced a computational method known as Multi-Omics Factor Analysis (MOFA), designed to uncover latent components within multi-omics datasets that reflect both biological and technical variability. Bayesian group factor analysis model that integrates multiple omics datasets by modeling them as a combination of shared latent factors. Each dataset is represented as a matrix decomposed into a product of a shared latent factor matrix and a modality-specific weight matrix, plus noise. The model applies sparsity-inducing priors to enable feature selection and to determine which latent factors are relevant for each data modality. Variational inference is used for scalable posterior estimation, allowing MOFA to handle missing data and uncover both shared and view-specific sources of variation in a fully unsupervised manner. Such integrated methodologies ease the exploration of information transfer among various layers of omics, thus bringing a gap between genetic make-up and observable traits.

Such widespread research suggests that changes in the cancer cells’ genomes significantly impact the clinical treatment efficacy of anticancer therapies. Mutations in the genome can also act as molecular markers to identify individuals who are at a more substantial benefit from certain treatments compared to others. To fully encompass the complexity involved in precision medicine, it is important to apply integrative analyses that genuinely integrate molecular data with treatment sensitivity.

The ultimate objective of precision medicine is to give the right drug to the right patient at the right time. However, the existence of intratumor heterogeneity makes it possible for different patients undergoing the same drugs to respond quite variably. Predicting the response of an individual patient to a given treatment or combination of treatments is one of the biggest challenges in oncology research. In an attempt to address this problem, AI-based methodologies are fast emerging. That can greatly reduce drug discovery into a supposedly more manageable resource for AI, putting the use of new medications into the spotlight.

DL models can take advantage of multi-omics data for cancer drug response assessment based on molecular profiles to improve the clinical relevance of precision medicine. In 2019, the architecture proposed by Chiu et al. (2019) is a feedforward deep neural network designed to predict tumor drug response (IC50 values) using integrated genomic profiles. It takes a combined input vector of gene expression, CNA, and mutation data, which is fed into multiple fully connected layers with ReLU activation and dropout for regularization. The final output layer uses a linear activation function to generate continuous IC50 predictions. The model is trained using mean squared error loss and optimized with Adam, outperforming traditional ML methods in predictive accuracy. These networks were subsequently integrated to provide a collective prediction of drug responses.

Wang et al. (2022) presents a DL framework that integrates five types of omics data—mRNA expression, gene mutations, CNA, RPPA, and metabolomics to predict anticancer drug responses found in the Cancer Cell Line Encyclopedia (CCLE) and the Genomics of Drug Sensitivity in Cancer (GDSC). The model uses graph-based embedding layers for omics data with known interactions (guided by the HINT interactome) and a dense embedding layer for metabolomics. These features are further processed through dense layers and integrated using an attention mechanism that assigns weights to each omics type based on its predictive value. The final output layer predicts drug response (IC50), enabling a biologically informed and data-driven approach to personalized cancer treatment. They implemented a graph embedding layer to incorporate interactome data and an attention layer to integrate distinct omics features, achieving a drug response prediction accuracy of 98%.

Almutiri et al. (2023) presented a novel approach that merges Bayesian Ridge Regression (BRR) with Deep Forest techniques to predict drug response. The model first integrates multi-omics data (gene expression, CNA, and single nucleotide variant) using BRR for feature selection and coefficient estimation. It then uses Deep Forest, which leverages Multi-Grained Scanning and Cascade Forest to capture local patterns in the data while progressively refining predictions through successive layers. This hybrid approach enhances prediction accuracy by integrating probabilistic modeling with efficient, computationally cost-effective DL methods, demonstrating superior performance root mean square error (RMSE) of 0.175, a Pearson correlation coefficient (PCC) of 0.842, and an R-squared value of 0.708) on the cancer cell line encyclopedia (CCLE) dataset.

Moreover, Malik et al. (2021) introduced a comprehensive methodology that encompasses multi-omics data, including CNA, mutations, methylation, miRNA, RNA, and protein expressions, to accurately assess survival outcomes and treatment responses in breast cancer patients. The model processes the data through separate streams for each omics type, followed by a fusion layer that combines the information. It consists of multiple hidden layers that learn complex patterns in the high-dimensional data, with an output layer dedicated to either survival prediction (regression) or drug response classification (categorical). The deep neural network is trained using cross-validation and evaluated with standard performance metrics to ensure robust predictions and insights for personalized treatment. They employed the Neighborhood Component Analysis (NCA) algorithm for supervised feature selection from multi-omics datasets sourced from The Cancer Genome Atlas (TCGA) and GDSC, demonstrating a 94% accuracy rate in classifying patients into distinct risk subtypes.

Other architectures, such as MOLI (Multi-Omics Late Integration) and AGMI, demonstrate the expanding role of late integration, graph neural networks, attention mechanisms, and explainable multi omics modeling in therapeutic prediction (Sharifi-Noghabi et al., 2019; Feng et al., 2021). MOLI (Sharifi-Noghabi et al., 2019), a deep neural network framework for the prediction of drug responses by integrating gene expression, DNA methylation, and somatic mutation data. MOLI utilizes modality-specific subnetworks for late integration to preserve biologically relevant information from individual omics layers, while improving predictive performance over single-omics models. But even with enhanced prediction accuracy, the framework is still very much based on retrospective datasets and lacks extensive external clinical validation. AGMI (Feng et al., 2021) is a framework based on an attention-guided graph neural network (GNN) for multi-omics drug-response prediction. AGMI is different from conventional neural networks because it encodes molecular interactions and attention mechanisms, which makes it possible to interpret relationships between molecules at the pathway level. Thus AGMI can be classified as biologically informed and interpretable precision medicine frameworks. (Munquad, 2024) reported an integrated framework combining network medicine and AI for subtype-specific drug response prediction in glioma. Subtype-specific disease modules were constructed, followed by DL–based drug response prediction using gene expression, mutation, and pharmacogenomic data. The framework demonstrated that distinct glioma subtypes exhibit differential drug sensitivities, supporting personalized precision therapy approaches. However, the study was limited by relatively small subtype-specific cohorts, and limited external validation across independent clinical datasets, which may affect model generalizability and robustness.

These predictive models of drug sensitivity by computation played an important role in the choice and selection of candidate compounds for preclinical investigation. By utilizing multi-omics data combined with existing knowledge on molecular interaction through AI, crucial targets for drugs that play an important role in the progression of cancer and can potentially be targeted by therapy may be identified. The further development of precision medicine and tailored treatment of cancer depends on predicting an individual’s response to drug. However, there are limited models for tissue-specific drug response prediction in glioma or lower-grade glioma (LGG) with independent external validation and biological interpretability. Moreover, drug sensitivity datasets with genomic information are usually highly imbalanced, which may influence the translational applicability and robustness of DL models. To overcome this, transfer learning is an appealing approach to address the data scarcity, in which the models are first trained on the large-scale preclinical datasets and then validated using the clinical data (Yang et al., 2025). These AI-based approaches are crucial for the development of clinically relevant drug response prediction models tailored to brain cancer and to translate in clinical settings.

## 6 Limitations and challenges in clinical implementation

The intersection of brain cancer genomics and deep DL is a dynamic and rapidly advancing research domain (Min et al., 2017). However, several challenges persist in creating effective diagnostic and therapeutic models. The application of DL to cancer multi-omics, methylation profiling, treatment response prediction, and survival modeling has been much improved, there are still several difficulties in clinical translation. A common problem in most investigations is the imbalance between high-dimensional omics features and relatively small cohort sizes. Many frameworks such as DeepGene, DeepOmix, CNA_origin, MethPed and glioma drug response prediction models were trained on limited patient’s cohorts or cancer cell lines leading to higher vulnerability to overfitting and worse model robustness across heterogeneous clinical populations. Furthermore, much research was predominantly based on retrospective TCGA-, GDSC- or CCLE-derived datasets with inadequate independent external validation, limiting confidence in the generalizability and repeatability across sequencing technologies, institutions and patient populations. Here we list primary obstacles in clinical translation:

- Interpretability Issues: One of the significant challenges associated with DL models is their lack of interpretability, often referred to as the “black box” problem. The complexity inherent in these models makes it difficult to understand the relationships between input data and resulting outcomes. In cancer research, there is a pressing need for biologically interpretable AI and DL algorithms to facilitate biomarker discovery and Clinical translatability (Ribeiro et al., 2016).
- Curse of Dimensionality: A significant challenge faced by AI applications in genomics is the curse of dimensionality. Genomic datasets typically exhibit high dimensionality, indicating that they contain a large number of features in comparison to a small number of samples (Clarke et al., 2008). This issue can be mitigated through variable selection methods (Hussain et al., 2024; Afshar and Usefi, 2020; Ghosh and Cabrera, 2022).
- Class Imbalance: Many DL models easily suffer from overfitting because the case of cancer datasets contains a significantly lower number of normal samples than those of cancer samples. Such a discrepancy hampers the functioning of ML and DL models in proper working unless sufficient samples per class are included in the training process. Combining datasets from various platforms and databases using appropriate normalization techniques can help address this challenge (Liu et al., 2023; De Angeli et al., 2022; Al-Stouhi and Reddy, 2016; Tasci et al., 2022).
- Data Heterogeneity: Such variability within cancer genomic data can lead to a varied performance in the DL model depending on the source of its data. Furthermore, genomic data is often produced with the use of different platforms such as microarray technology and next-generation sequencing. The high variability between the datasets collected with these two types of platforms can limit the development of robust DL models for specific cancer types (Goswami, 2020; Zhang et al., 2023).
- Generalizability: Computational models should be validated in a demographically and clinically diverse population to test their robustness. Model performance can be significantly impacted by variations in diagnostic tools, laboratory protocols, and standard treatment guidelines (Chekroud et al., 2024; Ly, 2024).
- Equity and inclusivity: The lack of minority representation in training datasets can reinforce therapeutic disparities. Inclusion of diverse populations in terms of ethnicity, socioeconomic status and geographic origin as well as the use of bias-detection frameworks and population-specific genomic variables should be incorporated to improve the fairness and clinical applicability of AI models (Yoo, 2026)
- Hyperparameter Tuning: Another challenging task while applying DL for cancer genomics applications involves fine-tuning model parameters. The DL architecture usually consists of several hyperparameters including batch size, momentum, and learning rate. Batch size refers to the count of training samples in each iteration; momentum helps determine the optimal training trajectory, and the learning rate determines the step size taken to minimize loss during training (Raiaan et al., 2024). Incorrectly setting these hyperparameters can lead to issues of underfitting or overfitting, negatively impacting model performance (Nematzadeh et al., 2022; Han et al., 2021; Smith, 2018).

Although overcoming some of these technical hurdles has resulted in the development of more precise and sophisticated multi-omics models, there are still a number of barriers that prevent their practical clinical use. A prime among them has been and still remains concerns around ethics, notably patient confidentiality, informed consent, and safeguarding against responsible misuse of highly sensitive genomic data. Ensuring the fairness, transparency, and population-level generalizability of AI models is central to preventing unintentional biases introducing potentially reinforcing inequities within healthcare (Mohr et al., 2024). Practical constraints also limit integration into routine healthcare practice. DL algorithms commonly demand great amounts of computational power, specific hardware, and experienced staff, which are not readily accessible within the majority of clinical environments. Second, the considerable costs of complete multi-omics assessment, including whole-genome sequencing and transcriptome quantification, impose great economic burdens on both institutions and patients. The rapid generation of genomic and multi-omics data also further complicates data storage, management, and security issues. Storage, processing, and upkeep of these enormous datasets require high-capacity storage systems, sophisticated cybersecurity infrastructure, and standardized data formats to allow for efficient sharing and integration between platforms (Koumakis, 2020). Additionally, adherence to intricate regulatory systems over the application of genomic data, including HIPAA and GDPR, presents added operational hurdles. These laws require robust data governance processes, secure sharing mechanisms, and open auditing systems (Williamson and Prybutok, 2024; Bonomi et al., 2020). Overall, while DL has revolutionary potential in cancer genomics, overcoming the ethical, practical, economic, infrastructural, and regulatory hurdles is necessary before the deployment of these technologies can be depended upon in actual clinical practice.

## 7 Conclusion and future scope

In this article, the DL models and their architectures applied in brain cancer genomics are discussed. DL holds considerable promise for assisting clinicians in avoiding human error and providing enhanced cancer diagnosis as well as analysis of complex data streams. It thus has emerged as a cutting-edge technology in cancer research. The review offers insights into different facets and applications of DL models, ranging from the diagnosis of brain cancer to the advancement of precision medicine.

The exceptional precision of deep learning approaches across diverse genomic data types indicates the potential to create robust models from the heterogeneous data typical of cancer patients. The DL models, which are summarized in this review, are mainly developed using data obtained from tumor tissues derived from brain cancers. The repeated sampling of brain tissue samples for continued generation and analysis of data is practically an unviable task. Therefore, DL developers and cancer researchers must pivot towards utilizing data derived from liquid biopsy samples obtained through non-invasive methods, such as blood, saliva, serum, and urine. This approach can facilitate early biomarker identification in brain cancer and simplify the collection of multiple time-point samples to assess patient responses to treatment.

The primary clinical diagnostic methods for brain cancer currently include imaging techniques, such as MRI and computed tomography (CT) scans, which reflect the anatomical features of the tumors. Combining DL methods with anatomical information from imaging analysis and genotypic information derived from genomic analyses could significantly advance the quality of diagnosis and treatment for brain cancer patients. Despite this potential, comprehensive multi-modal analyses that combine imaging and genomic data in the context of brain cancer remain largely unexplored. Further research in data integration and the development of multi-modal DL models could lead to substantial advancements in diagnosis and therapy. In this context, transformer-based models have recently emerged as a promising DL architecture in brain cancer research because of their ability to capture long-range dependencies and complex multimodal biological patterns. Unlike CNNs, self-attention architecture in transformers may facilitate efficient integration of genomic, histopathological, and radiological information. In neuro-oncology, transformer architectures have been increasingly used for glioma classification, tumor segmentation, and molecular subtype identification from MRI and histopathological data (Jin, 2026; Kumar and Jyoti, 2026). However, the application of transformer models in genomic data for brain cancer is still rather scarce.

Another emerging field is Multimodal foundation model, which can integrate heterogeneous data modalities, such as radiology, pathology, clinical information and molecular profiles, into a unified representation for precision therapy. A recent study by Fang, (2026) demonstrated the potential of multimodal foundation models for brain disease analysis through integrating large-scale imaging and clinical datasets to improve generalizability. The main focus of the study was multimodal brain imaging and clinical foundation model. But, future multimodal foundation models can integrate genomic and multi-omics data with pathological information to drive precision medicine applications.

Furthermore, the development of clinically interpretable and externally validated AI frameworks coupled with rigorous experimental and prospective clinical validation will be essential for the translation of current computational models into reliable tools for precision brain cancer diagnosis, prognosis and personalized therapy.
